# Should Artificial Intelligence Be Used for Physician Documentation to Reduce Burnout?

**DOI:** 10.34067/KID.0000000000000430

**Published:** 2024-03-25

**Authors:** Jing Miao, Charat Thongprayoon, Wisit Cheungpasitporn

**Affiliations:** Division of Nephrology, Department of Medicine, Mayo Clinic, Rochester, Minnesota

**Keywords:** nephrology

Physician burnout is a state of emotional, mental, and often physical exhaustion caused by prolonged exposure to stressors in the health care environment. While its exact prevalence remains undetermined, it is evident that burnout has become more common recently. About 63% of physicians in the United States feel burnout symptoms weekly.^1^ According to the 2023 Medscape report, burnout among nephrologists has increased from 40% to 44% over 5 years.^2^ The consequences of burnout extend beyond individual health, leading to increased risk of medical errors, decreased patient satisfaction, higher physician turnover, and overall a negative impact on the health care system's efficiency and effectiveness. A significant contributor to burnout is the overwhelming workload, marked by long hours, a high volume of patient care responsibilities, and administrative tasks. A notable aspect exacerbating workload intensity involves the extensive time dedicated to medical documentation. This not only encompasses the drafting of various notes, reports, and discharge summaries but also extends to managing communication through portal or In Basket message replies.^1^

The advancement of artificial intelligence (AI), particularly in the development of large language models (LLMs), offers significant potential to assist health care providers with medical documentation through its sophisticated language processing capabilities. Generative Pre-Trained Transformers-4, currently the most advanced iteration of ChatGPT developed by OpenAI, emerges as the most comprehensive and powerful generative model to date. This is attributed to its broad range of extensive training data, enabling it to engage in discussions across a vast array of disciplines and topics. In addition, its capabilities in image processing are particularly promising, especially for applications in the medical field.^3^ Using ChatGPT for physician documentation to reduce burnout presents an intriguing proposition, blending the potential for enhanced efficiency with the need for precision and reliability in medical records. Opinions on integrating AI into health care practices vary widely, from those asserting ChatGPT is not the solution to alleviating documentation burden on physicians^4^ to others who see its potential, “Hey chatbot, please generate a history and physical report for me.”^5^

Using AI models such as ChatGPT can improve the documentation process, which may subsequently reduce the workload for physicians. Several studies highlighted ChatGPT's ability to construct precise and effective discharge summaries and operative reports for ophthalmologists and plastic surgeons.^6,7^ Moreover, the inclusion of AI-created hyper-realistic visuals significantly improved the quality of these operative notes.^6^ A randomized controlled trial suggests that, even with the presence of certain inaccuracies, ChatGPT surpassed both dictation and typing methods in documenting the history of present illness, as measured by the Physician Documentation Quality Instrument score.^8^ In addition, a comparative study indicates that despite not being perfect, ChatGPT has improved the documentation of informed consent for surgical procedures. It has outperformed surgeons in terms of the thoroughness, precision, and the quality of descriptions regarding the benefits and alternatives of surgeries.^9^ Furthermore, a pilot study reported that evaluators favored the ChatGPT's replies to patient inquiries, deeming them of superior quality and exhibiting greater empathy compared with those provided by physicians.^10^

It is important to emphasize that ChatGPT's capabilities extend beyond creating discharge summaries, operative reports, question answering, and informed contents; it has a broad application across various aspects of medical documentation within the health care domain including nephrology (Table 1). ChatGPT provides substantial supports in health care documentation by organizing clinical records on the basis of health care professional inputs. It facilitates transcription process, transforming audio or handwritten notes into digital formats, and simplifies document creation through customizable templates for consent forms and other regulatory documents. In addition, it can summarize telehealth consultations for records, draft administrative correspondences, such as patient letters, and generate tailored patient education materials, ensuring clarity and comprehension for diverse patient groups.

**Table 1:** Potential applications of artificial intelligence such as ChatGPT in nephrology medical documentation

Application	Examples	Strategies to Improve ChatGPT's Capabilities
Clinical note creation	• Draft outpatient notes and tailored messages to patients between-visit care • Draft HPI, H&P examination notes • Draft consultation, progress, and transfer notes, discharge summaries • Draft procedure and dialysis notes • Draft multidisciplinary team meeting notes, prescription and medication management notes, laboratory and imaging interpretation notes • For kidney transplant, draft medical evaluation documents, insurance and financial documents, legal and consent forms, counseling and education records, post-transplant care plan	AI integration and customization • Incorporate ChatGPT within EHRs to enhance workflow efficiency and enable seamless access to patient records for automated document creation, while ensuring HIPAA compliance through deidentification of PHI. • Adapt ChatGPT to enhance its comprehension of medical terminology, documentation standards, and the unique requirements of individual health care provider practices or specialties Training and support • Provide comprehensive training for health care providers on how to use ChatGPT effectively, focusing on input accuracy, review processes, and data security practices • Ensure ongoing IT support to address technical issues, update AI models, and adapt to changing documentation requirements Pilot testing and incremental implementation • Start with pilot projects to test the effectiveness of ChatGPT in specific documentation tasks, allowing for adjustment and optimization before wider implementation • Gradually implement AI tools across different departments or documentation types Quality control and monitoring • Implement quality control processes where health care providers review and validate AI-generated documentation to ensure accuracy and compliance • Regularly assess the performance of AI tools in terms of accuracy, efficiency, and user satisfaction, making adjustments as needed Feedback and continuous improvement • Regularly gather feedback from users to identify challenges, areas for improvement, and additional training needs • Use feedback and performance data to refine AI tools, training programs, and documentation processes continuously
Inbox or In Basket management	• Prioritize patient messages • Generate draft responses • Edit physician messages to optimize communication, including for literacy appropriateness	
Medical transcription support	• Convert voice or handwritten notes from clinical setting (*e.g*., outpatient clinic, consultations, surgeries, and patient interactions) into typed text	
Education and research	• Generate patient education materials based on individual comprehension level • Assist in drafting case reports by structuring the narrative around the patient's presentation, diagnosis, treatment, and outcomes • Draft grant proposals, ensuring clarity and coherence in presenting objectives, methodologies, and expected outcome • Draft ppt for presentation	
Template generation	• Create customizable templates for common documentation needs, such as consent forms, referral letters, and prescription orders	
Administrative support	• Draft correspondence, such as letters to patients, appointment reminders, and communication with other health care facilities	
Telehealth consultation summaries	• Generate summaries of telehealth consultations, capturing key points and recommendations	

AI, artificial intelligence; EHRs, electronic health record systems; H&P, history and physical; HIPAA, Health Insurance Portability and Accountability Act; HPI, history of past illness; IT, internet; PHI, protected health information.

Strategic planning is imperative for the successful integration of ChatGPT into clinical practice, ensuring optimized outcomes (Table 1). First, given the inherent limitations of ChatGPT in adhering to the Health Insurance Portability and Accountability Act, it is imperative for health care organizations to deidentify Protected Health Information before utilization of such tools. A possible solution is the ChatGPT Enterprise, designed to meet the needs of organizations seeking advanced security and privacy measures. From a patient viewpoint, integrating AI into health care for clinical note generation presents several potential impacts regarding privacy, accuracy, and communication with providers. Ethical transparency about AI's use in health care is crucial for addressing patient concerns about the quality of care and data management. To build trust and maintain high standards in patient care, it is essential to disclose the use of AI, ensure Health Insurance Portability and Accountability Act compliance, and verify the accuracy of AI-generated documents by health care professionals ourselves.

Second, AI and LLMs, such as ChatGPT, are increasingly being integrated into health care systems. This incorporation can enhance workflow efficiency and documentation accuracy. Notably, Nuance Communication, a Microsoft subsidiary, integrated ChatGPT with its Dragon Ambient eXperience Express to automate clinical documentation. In addition, Microsoft's collaboration with Epic electronic health records uses generative AI to draft responses to patient messages. Moreover, Oracle Cerner's electronic health record system now features the Oracle Clinical Digital Assistant, which facilitates automated note-taking and health care actions, ultimately enhancing interactions between health care providers, patients, and their data. Notably, regarding the current internet (IT) infrastructure in health care, it varies widely across different institutions and geographies. To accommodate the shift toward AI-enhanced documentation and ensure smooth integration of ChatGPT, expanding and upgrading existing IT infrastructure is essential. This involves hardware, software updates, and investing in IT staff training to manage and support AI technologies.

Third, fine-tuning ChatGPT's understanding of medical terminology and documentation standards can optimize its functionality for health care providers, improving medical documentation accuracy. It is also crucial to train health care providers thoroughly on the utilization of AI tools, focusing on the precision of inputs, adherence to review protocols, and data security measures. Moreover, pilot testing and phased implementation enable the evaluation and refinement of AI tools before wider application. Establishing quality control protocols for health care providers to verify AI-generated documentation and regularly assessing AI tools for accuracy, effectiveness, and user satisfaction are essential. User feedback is crucial for the continuous improvement of AI applications, ensuring an adaptable and responsive AI integration strategy in medical documentation.

While the precision of ChatGPT and similar LLMs enhances with continuous advancements in methodologies, their application in health care, particularly in low-risk but high-potential areas, such as documentation, to alleviate workload, is noteworthy. Examples include drafting appeal letters for medication or test usage, summarizing laboratory results, or offering dietary advice. However, it is imperative for physicians, including nephrologists, to acknowledge that we take the ultimate responsibility for the accuracy of clinical records. Errors or hallucinations in AI outputs should not be blamed on the AI. Instead, we must view these tools as assistants that can enhance efficiency but necessitate vigilant supervision. In academic institutions, where the responsibility to cosign or attest to a trainee's documentation exists, this supervisory role parallels the necessity to provide constructive feedback to enhance LLMs. This involves a thorough examination of AI-generated contents, identifying inaccuracies, and aiding in the evolutionary improvements of these models. Using these AI tools like ChatGPT in clinical practice highlights the essential role of physician oversight and the continued value of human judgment in ensuring patient care documentation's integrity and reliability.
